# Editorial Brevities

**Published:** 1888-10

**Authors:** 


### EDITORIALS AND MISCELLANEOUS.

### EDITORIAL BREVITIES.

UNION PHARMACEUTICAL CO.—The advertisement of this excellent house in to day's journal will well repay perusal.

THE second triennial session of the International Congress of Hydrology and Climatology will be held at Paris the first week of October, 1889.

SCOTT & BOWNE.—See the new advertisement of Scott's emulsion in this journal. This preparation has a well deserved and established reputation.

MCARTHUR'S SYR: HYPOPHOSPHITES COM. is an excellent preparation. It is prepared especially for physicians and should be patronized by the profession. See advertisement of this excellent preparation in this journal.

MEDICAL BOOKS.—See the advertisement of Lester & Kuhrt in the line of medical books. They are clever business men, and keep all the standard medical books.

REMEDIES FOR RESPIRATORY AFFECTIONS.—We invite special attention to the new advertisement entitled, "Remedies for Respiratory Affections," on fourth cover page of this journal. The enterprising house of Parke, Davis & Co. always have something good to offer and something interesting to say.

DOCTORS' CASES, SADDLE-BAGS, INSTRUMENTS, ETC.—The house of I. Phillips on Marietta street is an interesting place to medical students and doctors. Surgical instruments of all kinds, pocket and dissecting cases in abundance. Call and see Mr. Phillips, 14 Marietta street.

THE SOUTHERN SURGICAL AND GYNECOLOGICAL ASSOCIATION.—Dr. Davis, the Secretary, writes that the meeting of the Association was not held in Birminghdm on the 11th, 12th and 13th of September as announced, but has been postponed till the first Tuesday in December, owing to quarantine against yellow fever.

PRUNO-PHOSPHORATED SYRUP.—This preparation is coming into notice as a remedy for pulmonary diseases. It claims to be a superior tonic to the nervous system and to the digestive apparatus. It is gotten out by the Union Pharmaceutical Company of New York. See their advertisement in this issue.

DR. J. B. DANIEL, druggist, of Atlanta, has on hand a large lot of surgical instruments of every variety, including also teeth extractors, etc., which he is selling off at exceedingly low figures. Mr. Daniel is an active and accommodating business man. His store is directly opposite the entrace to the Union Passenger Depot. He runs an advertisement in this journal. Call and see him or send your orders. Dissecting instruments dirt cheap.

MEDICAL LIBRARY FOR SALE.—The library of Dr. J. T. Suggs, of Texas, deceased, is offered for sale at very low figures. His wife, who is in needy circumstances, and entitled to the sympathy of the brotherhood, writes that the library is a good one, containing many valuable works and bound journals. It furnishes a good opportunity to medical students or practitioners to procure a library at low figures. Parties desiring to purchase should address Mrs. J. T. Suggs, Mount Pleasant, Titus county, Texas.

THE DENTAL SCHOOL OF ATLANTA COMPLIMENTED.—The American and Southern Dental Associations convened at Louisville, Ky., on August 28th.

The National Association of Dental College Faculties met at the Gault House on the 27th of August. On a call for the percentage of examinations in the different schools, that of the Dental Department of the Southern Medical College, at Atlanta, was found to be five per cent. higher than any other school. As a consequence this school was very highly complimented, and its Dean, Dr. L. D. Carpenter, was elected Vice-President for the ensuing year.

ANOTHER MEDICAL AND SURGICAL REPORTER.—The editor writes: In our issue of July 28, we called attention to the appearance of a new medical journal which had taken the name of this one. Assuming that this was an error not inconsistent with honest intentions, we expressed the hope that it would soon be corrected. Since then, however, the second number of that publication has appeared, bearing the title: THE TOLEDO MEDICAL AND SURGICAL REPORTER—the first two words printed small, and the part of the title which copies ours printed in large letters. This step on the part of the publishers, we think, justifies the belief that they hope to profit by assuming our name, and are not to be deterred from it by those principles which actuate honorable men.

